# Supplementary material for: Epidemiology of prediabetes mellitus among hill tribe adults in Thailand
Source: PLoS One. 2022 Jul 25;17(7):e0271900. doi: 10.1371/journal.pone.0271900 (PMC9312415; doi:10.1371/journal.pone.0271900)
Supplement: S1 Appendix — (DOCX) [file pone.0271900.s001.docx]

**Questionnaire of the project of assessing diabetes mellitus**

**Part I general information**

1. Sex

Male Female

2. Age……………………Year

3. Education

No-education

Primary school

High school

University degree

4. Tribe

Akha

Lahu

Hmong

Yao

Karen

Lisu

5. Religion

Buddhist

Christian

Other

6. Occupation

Unemployed

Agriculturalist

Trader or other

7. Annual income…………………….baht

8. Having debt

Yes

No

9. Marital status

Single

Married

Ever married

10. Number of family member.......persons

11. DM history of father

No

Yes

Do not known

12. DM history of mother

No

Yes

Do not known

**Part II** Health behaviors and substance use

1. Exercise

No

Sometimes

Regular

2.Alcohol use

Yes

No

3. Smoking

Yes

No

**Part III** Physical examination

Weight ………………………………………………....kg

Height…………………………………………..………cm

Blood pressure.................................................................mmHg

Total cholesterol........................................................mg/dL

LDL ………………………………………………... mg/dL

HDL………………………………………………...mg/DL

Triglycerides………………………………………..mg/DL

HbA1c………………………………………………..
