# Supplementary material for: Epidemiology of prediabetes mellitus among hill tribe adults in Thailand
Source: PLoS One. 2022 Jul 25;17(7):e0271900. doi: 10.1371/journal.pone.0271900 (PMC9312415; doi:10.1371/journal.pone.0271900)
Supplement: Attachment — Submitted filename: Response to reviewers .docx [file pone.0271900.s004.docx]

**Response to reviewers’ comments**

**Review Comments to the Author**

Please use the space provided to explain your answers to the questions above. You may also include additional comments for the author, including concerns about dual publication, research ethics, or publication ethics. (Please upload your review as an attachment if it exceeds 20,000 characters)

Reviewer #2: Thank you for inviting me for this review. This is an interesting community-based cross-sectional study, and the first hand in-field data collection was valuable and provided some insights into the prevention and epidemiological understanding of prediabetes mellitus in Thailand. I hope my comments below are useful considerations.

1. Could you please clarify when the survey was conducted and time span of this study?

: Data were collected between November 2019 and March 2020; please see the abstract and methods sections (page 6, lines 16-17).

2. Statistical analysis section:
• Correcting “SPS program” with “SPSS program”? A typo maybe.

: Thank you, it has been corrected.

• Please clarify/specify your multivariable logistic model. The author mentioned that age and gender were the only two controlled confounders. Based on the results, it seems other variables were also included/controlled in the final multivariable model, while only the significant four variables were reported. Please provide a complete list of variables that you had used for this model. Based on the authors’ description of previous studies, it seems BMI, alcohol consumption and other socioeconomic factors all have impacts on the outcome to certain level. Therefore, please provide the rationale about your variable selection, in terms of model performance, data quality and clinical meanings.

: We started with the univariate analysis by having one independent variable and dependent variable and set the significance threshold at α=0.05, and any variable with a p value equal to or less than 0.05 was considered significant. This step of the analysis was repeated until all independent variables were completed. Afterward, all independent variables were added to the model with the dependent variable, and the significance was assessed. The least significant variable (with the greatest p value) was removed from the model, and the Hosmer Lemeshow Chi-square test was used to assess the goodness of fit (nonsignificant). The process of testing the model was repeated by removing all nonsignificant variables in the model and showing that the Hosmer Lemeshow Chi-square test result was nonsignificant, which was the final model. However, before interpretation, age and sex were adjusted in the model to control their effects as confounding factors.

Of course, with the conditions (both exposures (independent variables) and outcomes (disease) examined at the same time) of the cross-sectional study used in this project, which is intended to assess the prevalence and predict the factors associated with the outcome, the association detected might not be fully accurate, similar to other stronger study designs that focus only on testing the association.

During the analysis, we examined all independent variables with the outcome (pre-DM) and retained some variables as the best predictors in the model before making the interpretation.

We have added the essential information to the statistical analysis section, page 6 line 32; and page 7 lines 1-3.

• Any sampling weights applied in your data analysis since you have surveyed different geographical locations (different hill tribes)?

: Thank you for the great comment. We used five villages from each tribe to select the participants for the study. All people who met the criteria and lived in one of the five selected villages for each tribe were invited to participate in the study. Finally, with the number of people living in each village, the proportion of participants from each tribe was still reflected in the study population:

Karen 16%, Hmong 14%, Yao 13%, Akha 29%, Lahu 16.8%, and Lisu 10.5%.

Even the actual population in the six different tribes are a bit different: in 2019, there were 749 hill tribe villages in Chiang Rai Province, Thailand, which included 316 Lahu villages (51,339 persons (26.5%)), 243 Akha villages (74,403 persons (38.5%)), 63 Yao villages (16,227 persons (8.4%)), 56 Hmong villages (33,478 persons (17.0%)), 36 Karen villages (7,933 persons (4%)), and 35 Lisu villages (9,632 persons (4.9%)) [12]. Page 3, lines 20-23.

: We have carefully considered this excellent point and found that the statistics shown in Table 2, particularly the CIs, had high power, which means that the sample size was large enough for testing the hypothesis and that the proportions could well reflect the different sizes of the populations of each tribe. However, during sample size calculation, we did not consider this idea, and it will be used in our next project to ensure that the final model can accurately reflect the hill tribe population. Thank you so much.

• In Table 2, could you please present the proportion of each factor for the patients with prediabetes mellitus and patients without prediabetes mellitus? Then we have a clear picture about how the demographical/socioeconomic and medical factors were distributed in each cohort, for instance, to see if the gender decomposition is similar in the patients with prediabetes mellitus versus the patients without prediabetes mellitus.

: Yes, please see columns 2 and 3, which have been changed to the % column. Then, we can see the proportion distribution in each factor between those who had pre-DM and those who did not.

• It seems the two cohorts (the patients with prediabetes mellitus versus the patients without prediabetes mellitus) were not propensity score matched/ approximately matched cohorts. Please include this as a limitation/address the possible impacts for your model.

: Thank you. We completely agree with you and have included this as one of the key limitations of the study.

3. Please check the English writing grammar.

: Thank you. The English has been checked by American Journal Experts (AJE) with reference no. 85D4-4E23-480D-5FA2-42C9 .

4. I noticed the author published a similar paper in 2018:

"Apidechkul T. Prevalence and factors associated with type 2 diabetes mellitus and hypertension among the hill tribe elderly populations in northern Thailand. BMC Public Health. 2018 Jun 5;18(1):694. doi: 10.1186/s12889-018-5607-2. PMID: 29871598; PMCID: PMC5989444."

: The two projects are different. The first project you mentioned, which was published previously in BMC Public Health, was designed to identify factors that contributed to DM and HT in the elderly population. In current project, however, we focused on people between 30 and 59 years old. The current project was performed after our first project was completed, and we extended our ideas in the second project. The first project was supported by the National Research Council of Thailand, while the second project was supported by the Health System Research Institute, Thailand (Grant No 61-027). Therefore, the two papers are different.

Could please also provide participants’ selection flowchart in current study to see how the weighting sampling method could be applied if that is possible? The reason to do this is that I am wondering if any geographical impacts applied/pre-exists for the prevalence of prediabetes mellitus. After reading this similar work, I gained some background information in your study location and it seems the different hill tribe had various prevalence of type 2 diabetes mellitus and hypertension, it made me pondering if that case was also applied for prevalence of prediabetes mellitus.

: Thank you for the comment. Please see Figure 1 on page 4.

Reviewer #3: This interesting study investigated the prevalence of prediabetes among subjects from six hill tribes in Thailand, which answered an important research question. The manuscript is generally well written and clearly presented. I only have a few comments for the authors to consider.

1. This study found that people having a normal total cholesterol level are more likely to have prediabetes than those having a high cholesterol level. This is controversial in the literature. Although the authors provided some discussion around this finding, I would like to recommend the authors add more discussion around the potential confounding and the relation between LDL-C, HDL-C, and total cholesterol. Please also consider adding a few references here.

: Thank you for the great comment. We have added information including references in this section; please see page 16, lines 12-16.

2. I am wondering why only age and sex were adjusted as covariates in the multivariable analysis. Since there are six tribes included in this study and the authors mentioned each of the tribes has its own culture, it seems tribe is one of the confounders. Please consider adding some explanations or discussions.

: Thank you for the great comment. From our literature review, we found that age and sex were major confounding factors in predicting diabetes and prediabetes.

: We also reanalyzed entire steps and used the Karen tribe as the reference (because Karen had the lowest prevalence) and found that two tribes, namely, Akha and Lisu, had significantly greater odds of developing pre-DM. Thank you so much for noting this. We have added this information to the discussion section as well.

3. In table 2, please consider providing all the odds ratios from multivariable analyses, even if some are not statistically significant.

: We have added all ORs in the multivariate model; please see table 2.

Thank you so much.

TK

Assist Prof. Dr. Tawatchai Apidechkul, MSc (Infectious Epidemiology), Dr. P. H (Epidemiology)

Dean, School of Health Science, Mae Fah Luang University

Director, Center of Excellence of Hill Tribe Health Research, WHO-CC

Former Hubert H Humphrey Fellow (2013-2014), Emory University

Global Health Delivery Intensive (Harvard School of Public Health)
